# Finite element analysis of different configurations of fully threaded cannulated screw in the treatment of unstable femoral neck fractures

**DOI:** 10.1186/s13018-018-0970-3

**Published:** 2018-10-29

**Authors:** Jiantao Li, Menglin Wang, Lianting Li, Hao Zhang, Ming Hao, Chen Li, Lin Han, Jianfeng Zhou, Kun Wang

**Affiliations:** 1 Department of Orthopaedics, Chinese PLA General Hospital, No. 28 Fuxing Road, Beijing, 100853 People’s Republic of China; 2 Department of Otolaryngology Head and Neck Surgery, Peking University Third Hospital, Beijing, 100191 People’s Republic of China; 3 Department of Orthopaedics, The Third People’s Hospital of Qingdao, No. 29 Yongping Road, Qingdao, 266041 People’s Republic of China; 4 Department of Orthopaedics, Tianjin Hospital, NO. 406 Jiefang Road, Tianjin, 300211 People’s Republic of China; 5 Graduate School of the Second Military Medical University, Shanghai, 200433 China; 6 Department of Emergency, Chinese PLA General Hospital, No. 28 Fuxing Road, Beijing, 100853 People’s Republic of China

**Keywords:** Unstable femoral neck fractures, Finite element analysis, Fully threaded cannulated screws, Partially threaded cannulated screws

## Abstract

**Background:**

In the present study, we evaluated the mechanical outcome of different configurations formed by fully threaded screws and partially threaded screws in the treatment of unstable femoral neck fracture.

**Methods:**

The Pauwels type III unstable femoral fracture and the models of the fully threaded screw and partially threaded screw were constructed in 3-matic software and UG-NX software respectively. We then assembled the different screw configurations to the fracture model separately to form the fixation models. After meshing the models’ elements, we used Abaqus software to perform the finite element analysis. Parameters of von Mises stress distribution on the screws, peak stress, displacement between fracture fragments, and model principal strains in cancellous bone were reported.

**Results:**

Our results indicated that the peak von Mises stresses of screws was concentrated in the middle surface of the screw near the fracture line in each group. Peak stress value of the implants was highest in the model of triangle with posterior single screw. And the lowest stress values were observed in the triangular model. Fully threaded screw in each group underwent the most stress while partially threaded screw underwent a little bit of stress. Lowest displacement was observed in the triangular model. The volume of bone susceptible to yielding in the femoral neck region was the lowest for triangular configuration.

**Conclusions:**

For unstable femoral neck fractures, superior results were obtained by stabilizing the fracture with triangular configuration formed by one superior partially threaded screw and two inferior fully threaded screws. This study will require clinical confirmation as to its practicality in the management of unstable femoral fractures.

## Background

Femoral neck fractures are relatively common which account for almost 50% of all hip fractures [1]. The treatment recommendations vary depending on the fracture pattern and the patient’s age [2]. Partially threaded cannulated screws (PTS) have remained a standard method for the fixation of femoral neck fractures in young patients for many years [3, 4]. The principle for this fixation technique is to enable healing by controlled fragment impaction across parallel placed screws and avoid nonunion or osteonecrosis caused by fracture gap [5]. However, loss of reduction after fixation by three PTSs of femoral neck fractures is reported to be up to 39% within the first three postoperative months [6].

Recently, fully threaded cannulated screws (FTS) have been raised regarding alternative ways for the fixation of femoral neck fractures and got the satisfied radiographic results and clinical outcome [7, 8]. But the optimal configuration of the screws is necessary to be investigated in order to guide the clinical practice. To our best knowledge, there are not any mechanical studies of finite element analysis test being published to compare the mechanical stability of the different configurations of screws as fixation methods used in unstable femoral neck fractures. Therefore, in an effort to shed more light on this issue, we design this research to evaluate the mechanical strength of the different FTS patterns in the treatment of unstable femoral neck fractures.

## Methods

**Fig. 1:**
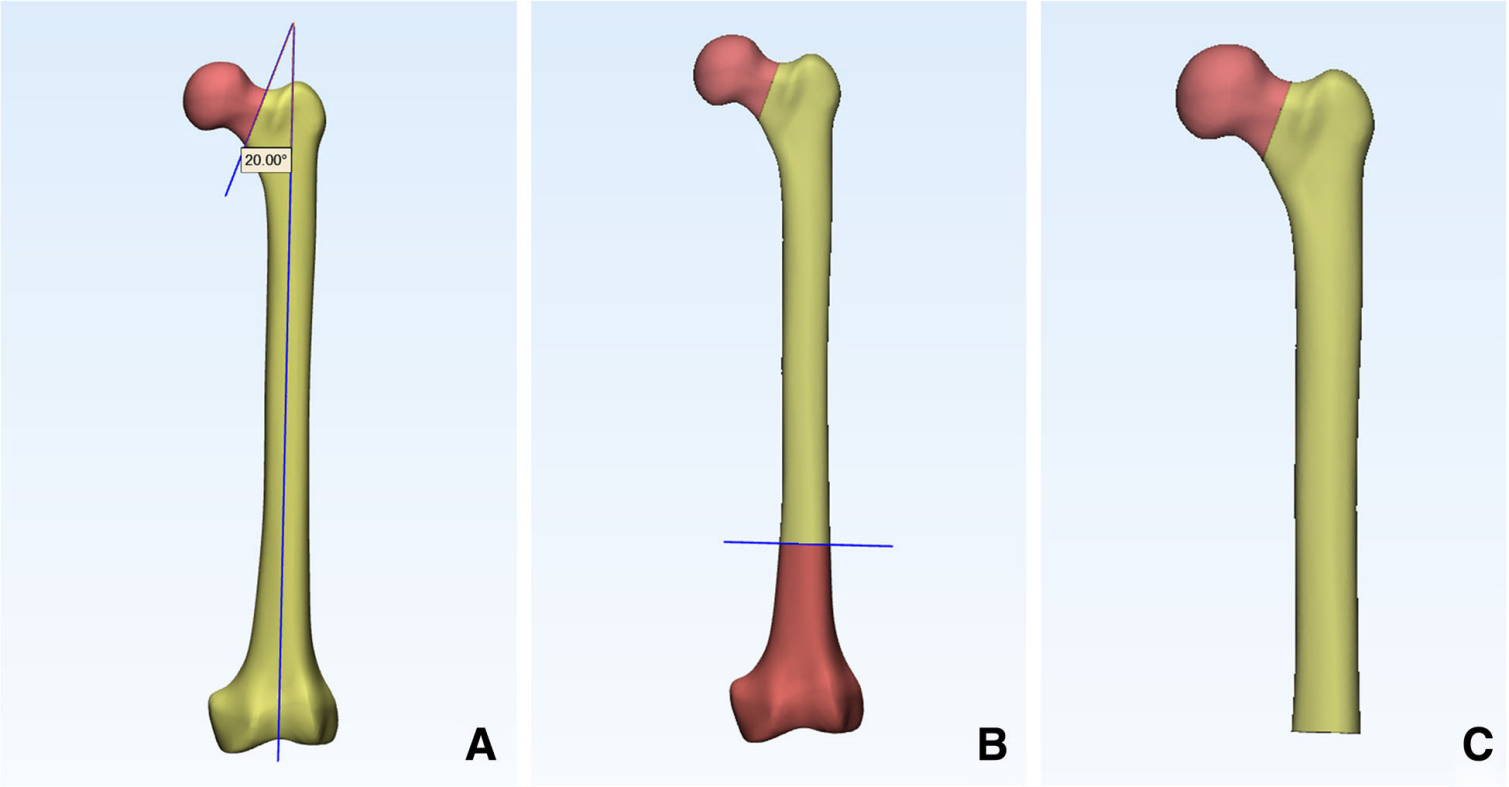
**a** A cutting plane across the center of the femoral neck at an angle of 20° with respect to the sagittal plane of the shaft axis was created. **b** A distal osteotomy plane was made 10 cm above the condyles of the femur. **c** The osteotomy model was created

The geometric model of the femur was employed from a three-dimensional model of a left fourth-generation composite femur (MODEL3405#, Pacific Research Laboratories, Vashon, WA). Then, we constructed the fracture model in 3-matic (Materialize, Belgian) to simulate the Pauwels type III unstable fracture [9]. We first created the femoral shaft axis, a cross which a sagittal plane was created. Then, we created a cutting plate that was across the center of the femoral neck at an angle of 20° with respect to the sagittal plane of the shaft axis. The femoral neck was cut by the cutting plane, simulating a Pauwels type III fracture (Fig. 1a). And a plane was made 10 cm above the condyles of the femur, which was the distal osteotomy plane (Fig. 1b). The osteotomy model was created (Fig. 1c).

**Fig. 2:**
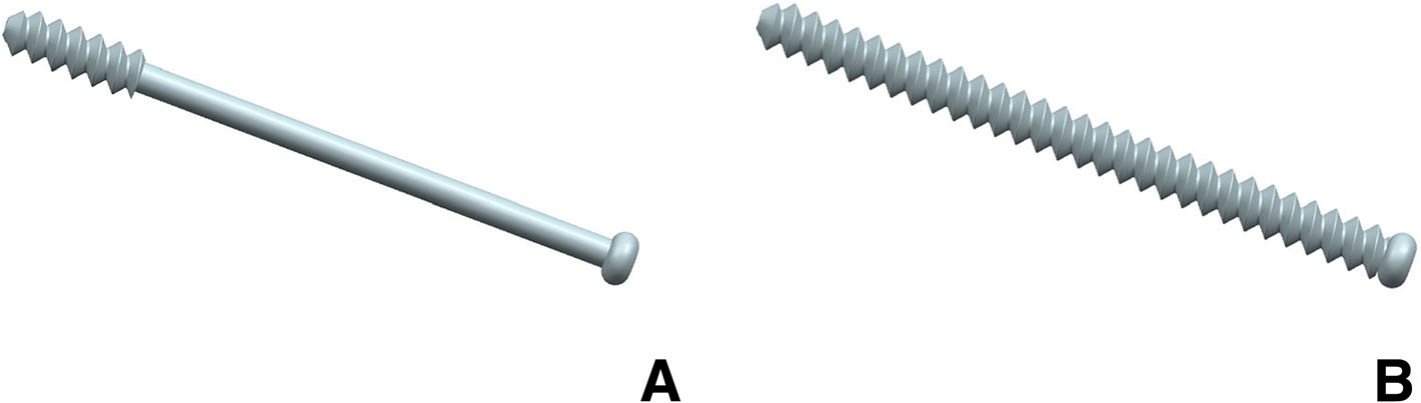
Geometric 3-D model of PTS (**a**) and FTS (**b**) were reconstructed using the software of Unigraphics NX 8.5

**Fig. 3:**
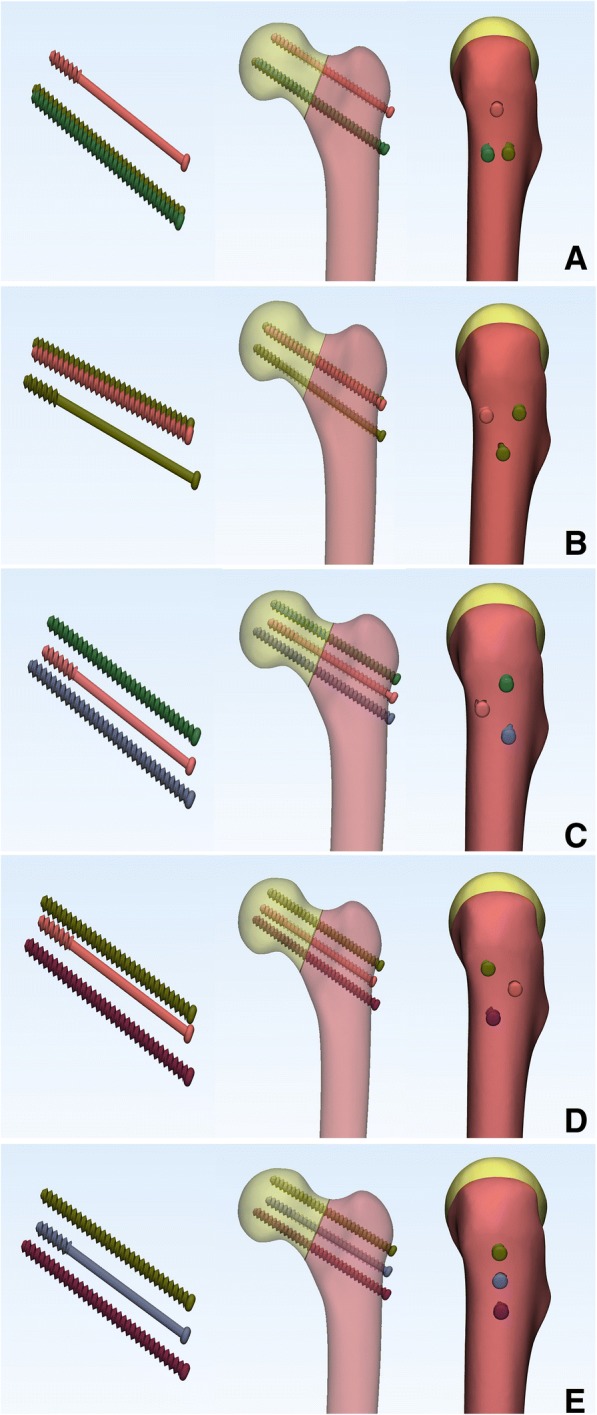
Assemblage of the screws and bones was accomplished in 3-matic to triangular configuration (**a**), inverted triangular configuration (**b**), triangle with anterior single screw (**c**), triangle with posterior single screw (**d**) and vertical configuration (**e**)

According to the manufacturer-provided engineering drawing, we reconstructed the geometric 3-D models of PTS (6.5 mm diameter and 16 mm thread length) and FTS (6.5 mm diameter and fully thread length) using the computer-aided design (CAD) software of Unigraphics NX 8.5 (Siemens PLM Software) (Fig. 2). The assemblage of the internal fixations and bones was accomplished in 3-matic to simulate the triangular configuration, inverted triangular configuration, triangle with anterior single screw, triangle with posterior single screw, and vertical configuration (Fig. 3). Fracture models were fixed by three parallel cannulated screws, out of which two were FTSs, one was PTS. The threaded tunnels left by screws were simulated through the boolean operation in 3-matic. All the models were meshed using the software HyperMesh 11.0 (Altair Engineering, Inc., USA).

The assembled 3D models were then imported into Abaqus (Simulia, France) to generate the finite element models. The bone model was assumed to be homogeneous, isotropic with linear elastic properties as reported by the manufacturer and previous studies [10–12]. Convergence tests were performed on triangular configuration models to ensure a fine enough element discretization for displacement analysis. The modulus of elasticity and the Poisson’s ratio were shown in Table 1. The screws were made of titanium alloy (Table 1). Tetrahedral 10-node elements (C3D10) were applied to the finite element models.

**Table 1:** Material properties used in the current study (titanium alloy, cortical, and cancellous bone)

Titanium alloy		Cortical bone		Cancellous bone	
E (GPa)	Poisson’s ratio	E (GPa)	Poisson’s ratio	E (GPa)	Poisson’s ratio
105	0.35	16.8	0.3	0.84	0.2

Frictional contact interactions were assumed between the different parts of the models. The threaded surfaces of screws were considered to be tie constraints (bone bonded to the screw). The interfaces between bone and PTS body were simulated by contact pairs with a friction factor of 0.3 [13]. Friction coefficients for bone-bone interaction was 0.46 [14]. All nodes on the surface of distal femur were constrained with 0 degrees of freedom to prevent rigid body motions during the analysis.

This study simulated the forces loading on the hip during the stance phase of walking. The FE models were applied a load (the force vector pointed laterally at an angle of 13° with the axis of the femoral shaft on the coronal plane, posteriorly by an angle of 8° with the shaft in the sagittal plane) of 2100 N corresponding to 300% body weight, and the force was introduced to the center of the femoral head [15]. In the analysis process, all forces applied to the proximal femur were divided into four steps to simulate the weight bearing process from partially to totally. Parameters of von Mises stress distribution on the screws, peak stress, displacement between fracture fragments, and model principal strains in cancellous bone were reported. A principal strain of 0.9% was taken as the yield strain value above which bone was susceptible to yielding in accordance with previously published data [16]. Regions characterized by strains larger than this value were assigned orange and red color to emphasize regions where bone tended to be susceptible to yielding.

## Results

The simulated thickness of the cortical bone in this study was 6 mm. The number of elements and nodes of the models was listed in Table 2.

**Table 2:** The details of models in this study

	Triangle	Inverted triangle	Triangle with anterior single screw	Triangle with posterior single screw	Vertical
Femur					
Elements	788,416	742,684	777,827	752,670	759,762
Nodes	161,809	152,348	159,510	154,261	155,471
Mesh size	Maximum: 3 mm; minimum: 0.5 mm				
PTS					
Elements	56,737	55,998	55,655	56,435	57,067
Nodes	101,995	100,341	99,884	100,885	101,736
Mesh size	1 mm				
FTS					
Elements	56,587	55,006	55,417	55,496	55,877
Nodes	101,756	98,673	99,370	99,481	100,004
Mesh size	1 mm				

### Von Mises stress distribution

**Fig. 4:**
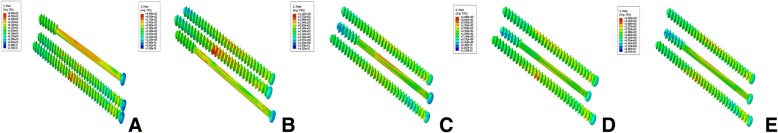
Stress distribution in five fixation configurations. **a** Triangular model. **b** Inverted triangular model. **c** Triangle with anterior single screw. **d** Triangle with posterior single screw. **e** Vertical model. Stresses appeared to be concentrated in the middle surface of the screw near the fracture line of each group

**Fig. 5:**
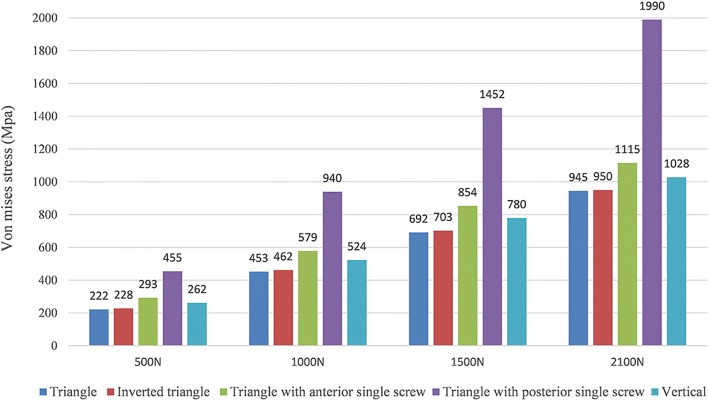
Graphic demonstration of the peak stress in five configurations under the increasing loads

**Fig. 6:**
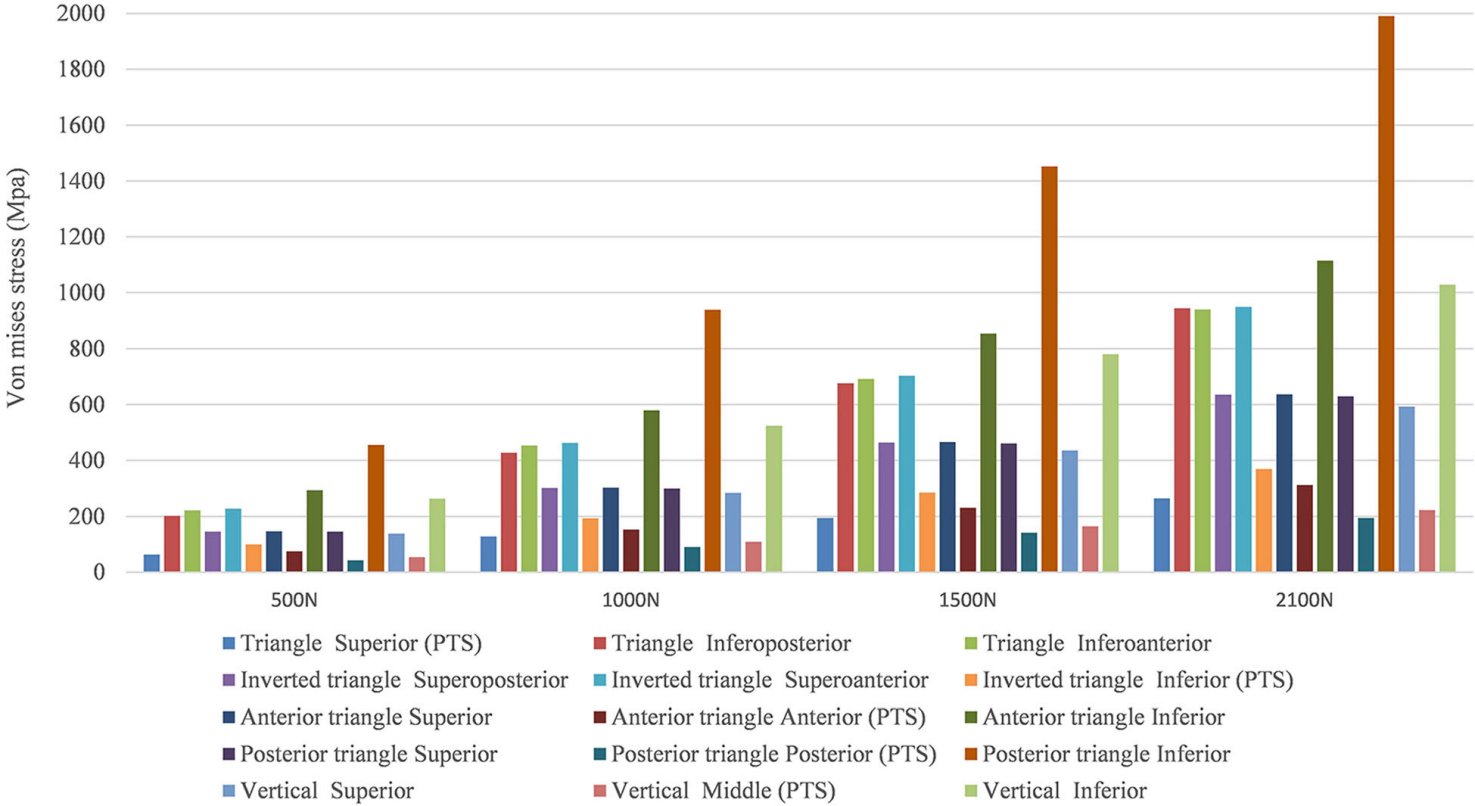
Graphic demonstration of the peak stress on each screw of different models under increasing loads

Differences of stress distribution were observed on the five configurations. Stresses appeared to be concentrated in the middle surface of the screw near the fracture line of each group (Fig. 4). Peak stress value of the implants was highest in the model of triangle with posterior single screw. And the lowest stress values were observed in the triangular model (Fig. 5). FTS in each group underwent the most stress while PTS underwent a little bit of stress (Table 3 and Fig. 6).

**Table 3:** Maximum stress (Mpa) on each screw of different models under increasing loads

	Triangle			Inverted Triangle			Triangle with anterior single screw			Triangle with posterior single screw			Vertical		
	S*	IP	IA	SP	SA	I*	S	A*	I	S	P*	I	S	M*	I
500 N	63	202	222	146	228	100	147	75	293	146	43	455	138	54	262
1000 N	128	427	453	301	462	194	302	153	579	299	91	940	283	109	524
1500 N	195	676	692	464	703	284	466	231	854	460	142	1452	435	165	780
2100 N	263	945	941	635	950	369	637	311	1115	629	195	1990	593	223	1028

*S* superior, *IP* inferoposterior, *IA* inferoanterior, *SP* superoposterior, *SA* superoanterior, *I* inferior, *A* anterior, *P* posterior, *M* middle

*Partially threaded cannulated screw

### The displacement between fracture fragments

**Fig. 7:**
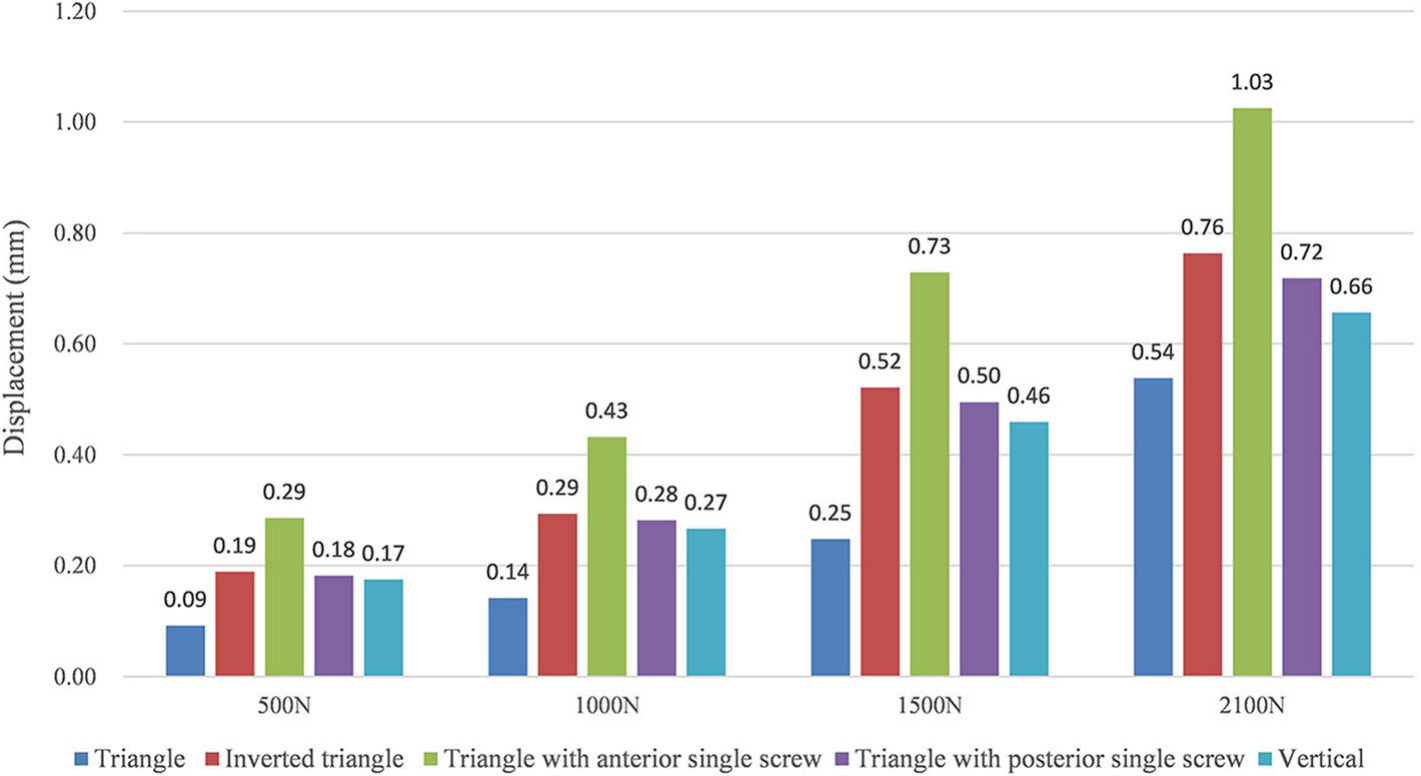
Graphic demonstration of the interfragmentary motions in five configurations under the increasing loads

Differences of fragment displacement were observed on the five configurations. Interfragmentary motions were calculated as the displacements between the two nodes on the proximal end of the fracture gap at the coronal view. Lowest displacement values were observed in the triangular model (Fig. 7).

### Maximum principal strains in the proximal fragment

**Fig. 8:**
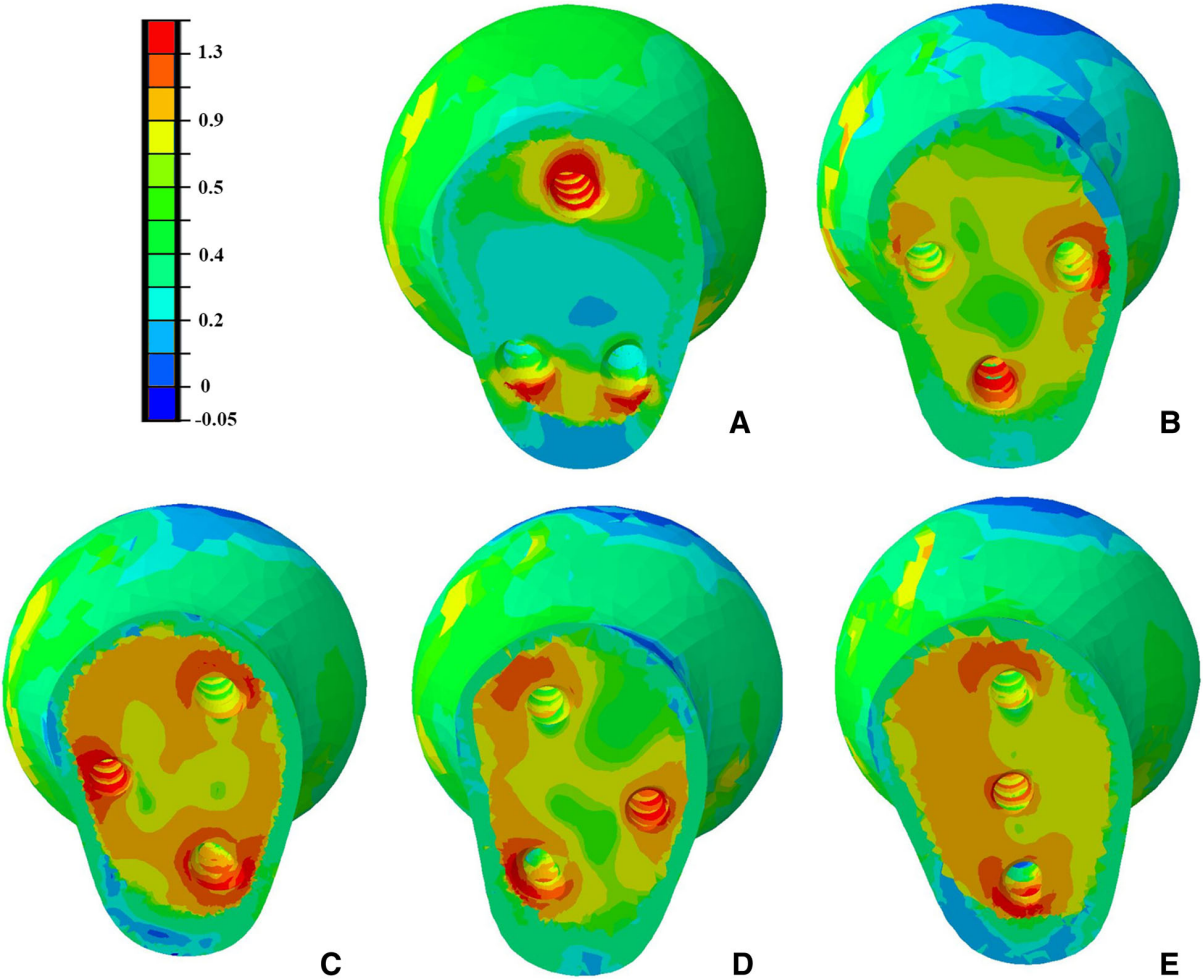
Diagrams showing the maximum principal strains plotted in percent with a yield strain cut-off value of 0.9%. Orange and red regions are susceptible to yielding strains above 0.9% and have a risk of cut-out. **a** Triangular model. **b** Inverted triangular model. **c** Triangle with anterior single screw. **d** Triangle with posterior single screw. **e** Vertical model

**Fig. 9:**
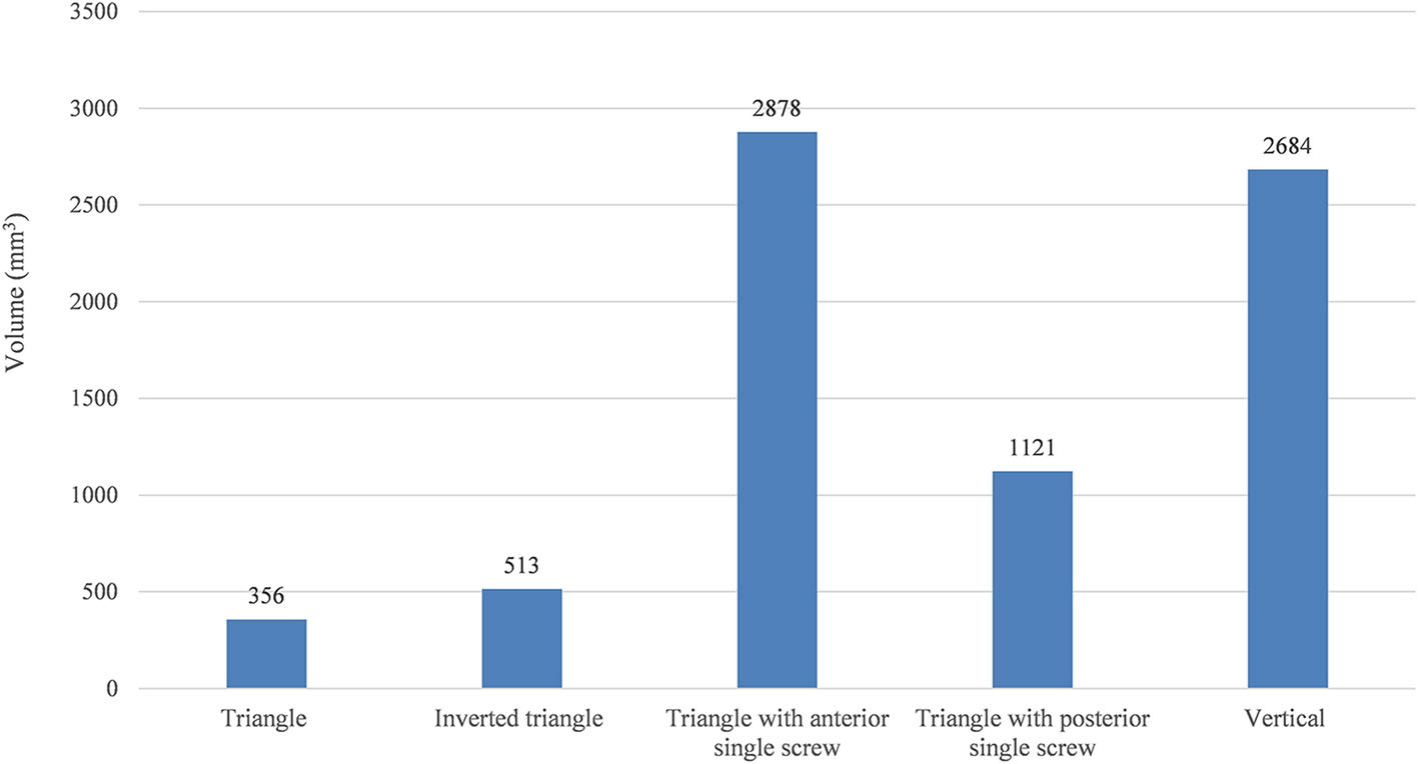
Volume of cancellous bone in the proximal fragment with maximum principal strains above yield strain value of 0.9%

Contour plots showing maximum principal strains in cancellous bone with a cross section through the femoral neck were shown in Fig. 8. This figure is based on the assumption that failure of screw cut-out from the head is likely to occur due to high strains in the weak region of the bone structure. The volume of cancellous bone in the proximal fragment with maximum principal strains above yield strain value of 0.9% was shown in Fig. 9.

## Discussion

In the present study, we investigated the mechanical distinction of different configurations combined by PTS and FTS in the treatment of unstable femoral neck fractures using the method of finite element analysis. This study demonstrated that triangular configuration formed by one superior PTSs and two inferior FTSs underwent lowest stress value, lowest displacement of fracture gap, and minimum yielding strains in the bone when compared to other configurations. Breakage of the inferior FTS in posterior configuration model may be more likely than other models. And configuration of anterior triangle and vertical screws may tend to have a risk of cut-out.

Several studies have compared the use of different PTS configurations for the fixation of femoral neck fractures [3, 17–20], but few have been published to compare the mechanical stability of the different screws configurations used in the unstable femoral neck fractures. Schaefer et al. [21] demonstrated that replacing the posterior PTS with a FTS in femoral neck fracture with posterior comminution showed a potential benefit compared with the three PTS fixation models. Zhang et al. [7] concluded that using the triangle configuration of a PTS plus two headless FTSs improved the outcome of unstable femoral neck fracture patients compared to those using PTS alone clinically and biomechanically. And Weil et al. [8] demonstrated a significantly decreased rate for femoral neck shortening after cannulated screw fixation when FTSs of inverted triangular configuration were used compared to PTSs. And the mechanical results of this present study can provide some guidance to its application in clinical practice. As shown in Figs. 4 and 5, under the axial loading, triangular screws underwent the lowest stress when compared with other four configurations. This can be explained by the fact that the triangular screws provide a better anchorage than other fixation types, thereby carry lower loads. Figure 7 illustrated that the triangular screws construct provided the greatest stability to the fracture fragment since the minimum displacement of the fragment gap was achieved in the triangular configuration under the increasing loads. As to unstable femoral neck fractures, with higher shear angle and greater shear force, postoperative complications like fixation failure, nonunion, and avascular necrosis were with high incidence [22, 23]. Heightened stability could improve femoral head blood flow leading to a decrease in avascular necrosis and union complications and was the better option in the treatment of the displaced femoral neck fractures [24]. Figures 8 and 9 showed the volume of the bone susceptible to yielding in the femoral head region, which indicated that the anchorage of the triangular screws was less likely to be involved in cut-out and was more stable. The stability of the screws within the head depends on an adequate anchorage in bone structure.

PTS has been introduced for the treatment of femoral neck fractures for many years, with advantages of less tissue invasiveness, less blood loss, shorter hospital stay, and shorter operation time [3, 25]. However, the vertical femoral neck fractures with a higher “shear angle” are so unstable that they have a higher rate of fixation failure and nonunion. The part-thread design of PTS made it obtain infinite pull strength by countering against the fragment, but with increasing the rate of screw withdrawal in the vertical fracture. And the full-thread design of FTS made it obtain more stable support to counter against the shear force of the vertical fracture, but without any pull strength to hold the fragment. The combination of the two types of screw used in the vertical femoral fractures could work. Difference mechanism made the PTS and FTS disperse different stresses. As shown in Table 3 and Fig. 6, the FTS in each group underwent the most stress while PTS underwent a little bit of stress. In the present study, we tended to use one PTS to provide the fragment compression and prevent gapping. And the function of two FTSs was to maintain the fracture reduction and resist the shear force. The results of finite element analysis indicated that the triangular configuration showed mechanical advantage compared with the other configurations.

No experimental validation was conducted, which clearly is a limitation. However, our aim was to examine trends rather than absolute values. In this respect, the lack of experimental validation is justified. A previous experimentally validated numerical study [10–12] employed the same loading and boundary conditions as our study. And more real biomechanical tests and clinical trials are needed to overcome the limitations of our study. Despite these limitations, this study is the first finite element analysis study to compare the mechanical efficiency of five different configurations combined by PTS and FTS for the treatment of unstable femoral neck fractures. Besides, we also simulated threaded tunnels of every screw in our models, with the mesh refinement around the areas which brought the parameter level to a more realistic value.

## Conclusions

In conclusion, the mechanical behaviors of five different screw configurations used to stabilize unstable femoral neck fractures were compared using finite element analysis. For unstable femoral neck fractures, superior results were obtained by stabilizing the fracture with triangular configuration formed by one superior PTS and two inferior FTSs when compared with other configurations of two FTSs and one PTS. This study will require clinical confirmation as to its practicality in the management of unstable femoral fractures.

## Abbreviations

CAD: Computer-aided design
FTS: Fully threaded cannulated screw
PTS: Partially threaded cannulated screw
